# How essentialist reasoning about language acquisition relates to educational myths and policy endorsements

**DOI:** 10.1186/s41235-023-00481-2

**Published:** 2023-05-05

**Authors:** Xin Sun, Shaylene E. Nancekivell, Priti Shah, Susan A. Gelman

**Affiliations:** 1 University of Michigan, Ann Arbor, MI USA; 2 Department of Psychology, University of British Columbia, 2136 West Mall, Vancouver, BC V6T1Z4 Canada; 3 University of Manitoba, Winnipeg, MB Canada

**Keywords:** Language acquisition, Psychological essentialism, Bilingualism, Multilingualism, Educational policy, Neuromyths

## Abstract

How people conceptualize learning is related to real-world educational consequences across many domains of education. Despite its centrality to the educational system, we know little about how the public reasons about language acquisition, and the potential consequences for their thinking about real-world issues (e.g., policy endorsements). The current studies examined people’s essentialist beliefs about language acquisition (e.g., that language is innate and biologically based), then investigated how individual differences in these beliefs related to the endorsement of educational myths and policies. We probed several dimensions of essentialist beliefs, including that language acquisition is innate, genetically based, and wired in the brain. In two studies, we tested specific hypotheses regarding the extent to which people use essentialist thinking when reasoning about: learning a specific language (e.g., Korean), learning a first language more generally, and learning two or more languages. Across studies, participants were more likely to essentialize the ability to learn multiple languages than one’s first language, and more likely to essentialize the learning of multiple languages and one’s first language than the learning of a particular language. We also found substantial individual differences in the degree to which participants essentialized language acquisition. In both studies, these individual differences correlated with an endorsement of language-related educational neuromyths (Study 1 and pre-registered Study 2), and rejection of educational policies that promote multilingual education (Study 2). Together, these studies reveal the complexity of how people reason about language acquisition and its corresponding educational consequences.

## Background

Decades of scientific research have been devoted to understanding how children acquire language so efficiently and quickly. This work has spawned accounts ranging from more empiricist, domain-general learning theories to more nativist, domain-specific proposals inspired by Chomsky’s (1980) notion of a ‘language acquisition device.’ Of course, most scholars agree that both nature and nurture are important to some degree (Granena, 2013; Kidd et al., 2018; Wulff & Ellis, 2018). Less is known about how lay people reason about language acquisition, and how their conceptualizations of language acquisition may influence their views on real-world developmental and educational issues, such as their willingness to endorse bilingual policies in schools.

The present study aims to characterize lay individuals’ beliefs about the nature of language acquisition, and the potential consequences of their beliefs. We use psychological essentialism as the theoretical framework to assess individual differences in people’s reasoning about language. Psychological essentialism refers to the belief that members of a category share underlying properties that cannot be directly observed (Gelman, 2004; Haslam et al., 2000). In the case of psychological traits, taking intelligence as an example, an essentialist interpretation would be that one’s intelligence is determined at birth, has a substantial biological basis (e.g., genetics), and is relatively unaffected by life experience. Relying on this framework, we aim to characterize the breadth of people’s thinking about language by probing to what degree people essentialize different aspects of language acquisition, such as the ability to learn one versus multiple languages. We build on prior work demonstrating the importance of characterizing lay beliefs or intuitive theories of cognitive capacities (e.g., intelligence, Dweck, 1999). Our second goal is to investigate the potential consequences of any variability in people’s reasoning about language acquisition. Namely, we are interested in whether the degree to which individuals hold language essentialist interpretations is associated with the endorsement of language-related neuromyths as well as education policies. Indeed, some have speculated that one reason for opposing multilingualism policies may be the erroneous belief that dual language exposure could delay first-language acquisition (Espinosa, 2015; Hakuta, 1999; Pettito, 2009).

To ground our investigation, we next review what is known about people’s conceptions of language and language learning. Existing work on language learning and beliefs has taken three general approaches. The first has focused on understanding the “language mindset” in adults. The idea of mindset taps into people’s reasoning about the malleability of psychological characteristics: for example, individuals with a growth mindset believe that certain characteristics are malleable and can be developed, whereas those with a fixed mindset consider these characteristics to be stable traits that cannot be improved (Dweck, 1999). Using this framework, prior work has found that college students primed with growth mindsets about language were more likely to hold mastery-oriented goals and were more determined to learn a second language (Lou & Noels, 2016, 2020). Other work diving deeper into language mindsets has found that there are likely two distinct but complementary types: general language intelligence beliefs and second-language aptitude beliefs (Lou & Noels, 2017). “General language intelligence beliefs” assessed people’s beliefs regarding language acquisition (unspecified as to whether it is first-, second-, and/or multiple) as dependent on fixed capacities and unable to be developed, whereas “second-language aptitude beliefs” specifically referred to whether *second* language learning has these qualities. Results showed that these types mapped onto distinct factor structures, and that participants held distinctively more growth mindsets regarding second-language learning than language learning more broadly (Lou & Noels, 2017). In sum, this work suggests that how people reason about language likely differs for general versus second language learning in meaningful ways.

A second approach to investigating how people reason about language learning has focused on the developmental origins of “linguistic essentialism” (Byers-Heinlein & Garcia, 2015; Kinzler, 2020, 2021). Linguistic essentialism refers to the belief that individuals are naturally predisposed to speak their native language, due to something innate and fundamental in themselves (Kinzler, 2021). For example, it may entail the belief that ethnic Koreans are born to learn Korean due to something innate and biologically based. In fact, many children believe that a baby born to English-speaking parents and adopted by parents who speak another language will speak English when they grow up (Byers-Heinlein & Garcia, 2015; Dautel & Kinzler, 2018; Hirschfeld & Gelman, 1997). This body of work provides empirical support for the idea that children hold an essentialist way of reasoning about one’s native language. Prior research has not yet directly examined these beliefs in adults, but there are reasons to suspect that linguistic essentialism may not be limited to young children (e.g., Kinzler, 2020, 2021; Pinker, 1994). For example, anthropologists have observed that people from some Kenyan communities consider their language as intrinsically tied to their ethnic group and what distinguishes them from other outsider groups—in other words, they view the language people speak as an indicator of their essence (McIntosh, 2005). This work suggests that some individuals may hold essentialist beliefs not only about language as a general capacity, but also specific languages learned in childhood.

A final approach involved using qualitative interviews to assess parents’ and teachers’ intuitive theories about language development and learning. These studies suggest that people’s views regarding early language development may relate to their choices and practices of language instructions for children (Pettito, 2009; Pettito et al., 2001). For example, in one interview with parents whose children had language difficulties, parents who attributed these difficulties to ‘child’ factors (for example, the child themself is not ready to speak) were less likely to accept speech-language therapy services for their child (Głogowska, 1998). Similarly, when recruiting participants for a bilingualism study, Pettito et al. (2001) found that some immigrant parents chose not to raise their children to be bilingual because they believed children are naturally prepared to learn only one language, and therefore simultaneous bilingualism may cause confusion. However, these studies did not differentiate essentialist vs. non-essentialist factors, and research is needed for a more systematic understanding of how individuals’ reasoning about language may yield educational consequences.

Across the three approaches described above, a few patterns are suggestive. First, individuals make use of diverse causal theories to reason about language acquisition, and these interpretations may differ with regard to which components of essentialist reasoning people consider. For example, Lou and Noels’ work (2016, 2017, 2020) taps into people’s reasoning about the *stability* of language and second language learning, whereas Kinzler’s review (2021) looks at how people reason about the *innateness* of the native language. Second, qualitative interviews with parents and teachers suggest that how people reason about the nature of language learning may influence their thinking about language education. For instance, malleability beliefs were associated with personal motivation and choices in language classes (Lou & Noels, 2020). Nonetheless, it remains an untested question whether these essentialist interpretations of language have broader educational consequences, such as holding erroneous beliefs about how language develops (Pettito et al., 2001, 2009). To our knowledge, no prior studies have explicitly specified the full complement of these beliefs, the relations amongst them, and how they relate to potential real-world consequences such as endorsements of educational issues.

### The present studies

In the present studies, we present a unified framework to address people’s essentialist beliefs about language. We specifically examined three subtypes of language essentialism: specific language essentialism, first language essentialism, and multiple language essentialism. Specific language essentialism is the belief that individuals are predisposed to speak the language of their birth parents, indicating that this is an innate, biologically based capacity (as outlined by Kinzler, 2021). First language essentialism is the belief that there are individual differences in a person’s capacity to learn their first language, whereby some people pick up their first language more readily than others, and that these differences are innate and biologically based. Multiple language essentialism is the belief that there are individual differences in a person’s capacity to learn multiple languages, whereby some people pick up multiple languages more readily than others, and that these differences are innate and biologically based (closely tied to Lou and Noels’ (2017) work with a mindset framework on second-language learning).

We hypothesized that these aspects of essentialist reasoning may be important for how people reason about two education-related consequences: neuromyth beliefs and policy endorsements about multilingualism and multilingual education. We chose these as the outcomes for several reasons. First, prior research has found that people’s essentialist reasoning aligns with how they reason about neuromyths. For example, Nancekivell et al. (2020) found that individuals with an essentialist view on learning styles (a common erroneous belief that people learn better when they receive instructions in their dominant way of learning) are also more likely to believe that different learning styles lead people to use different brain regions to learn. Second, debates over multilingual education include language-related folk beliefs (i.e., the claim that children are not ready for early bilingual exposure; Hakuta, 1999; OECD, 2007). Finally, both neuromyth beliefs and policy endorsements are critical to real-world educational practices and policy-making, and therefore, a deeper understanding of how they are related to individuals’ theories about language acquisition will yield meaningful implications for education. To our knowledge, this investigation is the first to use psychological essentialism to study people’s beliefs about language acquisition, as well as their consequences.

Study 1 adapted survey measures from the psychological essentialism literature (e.g., Gelman et al., 2007; Nancekivell et al., 2020) to probe the three subtypes of language essentialism listed above, and further explored how these essentialist interpretations may be associated with neuromyth beliefs about multilingualism documented in the literature (Crawford, 1997; Garrity et al., 2019; Horvath et al., 2018; OECD, 2007). The pre-registered Study 2 further examined the implications of engaging in essentialist reasoning about language, by means of two sets of investigations: first, a replication of Study 1, and second, an examination of potential associations between essentialist reasoning and policy endorsements regarding multilingualism and multilingual education.

## Study 1

Study 1 aimed to (1) test people’s essentialist beliefs about language, and (2) explore how such beliefs are associated with multilingualism-related neuromyths. We probed language essentialism using an adapted version of the switched-at-birth paradigm (Heyman & Gelman, 2000; Hirschfeld, 1995; Moya et al., 2015; Sun et al., 2021; Taylor et al., 2009) as well as a language essentialism questionnaire (adapted from Nancekivell et al., 2020). In the classic switched-at-birth paradigm, participants were told about two babies who were born to two families but were switched at birth. Parents of the two families were substantially distinct in some trait(s), such as intelligence. Each baby, therefore, had their biological and adoptive parents differing in such traits. Participants were asked to rate the extent to which the baby would be more like their biological or adoptive parents on that trait. As a reliable measure, the switched-at-birth paradigm was widely used in child and adult studies to probe the variability of essentialist reasoning (Gelman et al., 2007; Moya et al., 2015).

The language essentialism questionnaire was built on previous essentialism questionnaires that measured different psychological constructs (e.g., learning style endorsement, Nancekivell et al., 2020). It had three subscales, measuring specific language essentialism, first language essentialism, and multiple language essentialism, respectively. Each subscale had items tapping into four aspects of essentialism: heritability, innateness, early emergence, and biological instantiation (Gelman et al., 2007; Nancekivell et al., 2020). The first-language and multiple-language essentialism subscales also included an additional aspect, namely, natural ability, whereas this aspect did not apply to the construct of specific language essentialism. Item descriptions were maximally matched in their wording across the three subscales.

Neuromyth beliefs about bilingualism were measured by a questionnaire adapted from prior studies (Crawford, 1997; Garrity et al., 2019; OECD, 2007). The questionnaire items included widely held ideas about bilingual learning that were debunked by scientific evidence (Horvath et al., 2018; OECD, 2002, 2007), for example, “The first language must be spoken well before the second language is introduced.” This myth assumes that successful bilingual speakers must learn their two languages in sequential order. On the contrary, much research indicates that children exposed to two languages in early childhood are able to achieve monolingual-like proficiency and their languages can interact and create positive transfer with each other (e.g., Chung et al., 2019; Sun et al., 2022). To ensure that any findings might be due to a general tendency to reject or accept all assertions, the questionnaire also included non-myth items.

## Method

### Participants

Participants included an undergraduate student sample (*N* = 386, *M(SD)*age = 18.83(1.10)) and a general adult sample (*N* = 340, *M(SD)*age = 33.22(11.37)), to assess the generalizability of these viewpoints. Student participants were recruited from the introductory psychology subject pool from a U.S. Midwestern public university and were given 0.5 credit hours for completing the study survey. The student sample demographics were as follows: gender, 63.5% female, 36.2 male%, and 0.3% not shared; race, 61.1% white, 29.3% Asian, 4.4% Black, 4.4% Latinx, and 0.3% Native American; 89.3% growing up in the US; and 39.1% self-reported as bi-/multilingual. The general adult sample was collected from Prolific, and each participant was given $3.00 for completing the same survey. The sample demographics were as follows: gender, 53.5% female, 45.6% male, and 0.9% non-binary; race, 72.6% white, 12.4% Asian, 6.8% Black, 6.5% Latinx, 1.5% Native American, and 0.3% Native Hawaiian or Pacific Islander; 99.4% growing up in the US; 24.7% self-reported as bi-/multilingual; and 50.9% holding at least a bachelor’s degree. All participants had a US IP address. An additional *N* = 43 undergraduate students and *N* = 10 adults were removed from the final sample because they failed to complete all the attention check items (see below). Data will be available on OSF upon publication.

### Procedure

Participants completed a survey with four blocks: Block 1 included the switched-at-birth scenario and questions; Block 2 included the language essentialism questionnaire; Block 3 included the neuromyth questionnaire; Block 4 asked for demographic information. There were also four attention check items (e.g., “Please choose Strongly Agree to this item.”), one per questionnaire block. The block order was fixed, and the item order was randomized within each block or subscale. The full survey can be found in Supplement 1.

### Measures

#### Switched-at-birth questions

In the switched-at-birth scenario, participants were told about baby Z who was born to Korean monolingual parents, then adopted by American parents in the US at birth. Baby Z’s adoptive family only spoke English and therefore Z grew up in an English-only environment. When Z went to high school, they started to take Korean classes. Participants were asked to predict the extent to which Z would find it easier to learn Korean than other students in the class in three aspects: picking up a native-like Korean *accent*, learning Korean *vocabulary*, and learning Korean *grammar*. The predictions were answered on a 6-point Likert scale (strongly disagree to strongly agree), and a higher value indicated a stronger essentialist view. The scenario was modelled on those from other studies (Gelman et al., 2007; Sun et al., 2021) and demonstrated high reliability (Cronbach’s alpha = 0.95 for both samples).

#### Language essentialism Questionnaire

The language essentialism questionnaire had 14 items (see Table 1) with three subscales: specific language essentialism, 4 items; first language essentialism, 5 items; and multiple language essentialism, 5 items. All subscales tapped into four dimensions of essentialism: heritability, innateness, early emergence, and biological instantiation; and the first and multiple language essentialism subscales each had an additional item for the dimension of “natural ability.” All items asked participants to rate the extent to which they agreed or disagreed with a statement. For each subscale, items were answered on a 6-point Likert scale (1-strongly disagree to 6-strongly agree), and a larger value indicated stronger essentialism. Note that due to the experimenter’s mistake, the student sample received a 5-point scale (1-strongly disagree, 3-neutral, 5-strongly agree). The questionnaire demonstrated high reliability (Cronbach’s alpha = 0.84 for the student sample and 0.87 for the adult sample).

**Table 1:** Language essentialism questionnaire items

Item label	Item wording	
*Specific Language essentialism*		
Genetically predisposed	People are genetically predisposed to learn the language(s) that their biological family speaks more easily than to learn other languages	
Brain	People’s brains are naturally tuned to learn the language(s) that their biological family speaks more easily than to learn other languages	
Determined at birth	People are born with the ability to more easily learn the language that their biological family speaks than to learn other languages	
Inheritable	A person inherits the ability to learn the specific language(s) of their biological parents (i.e., the language their biological parents speak)	
*First Language essentialism*		
Gene	In the future, scientists will be able to determine a person’s ability to learn their first language by examining their genes	
Brain	There are consistent differences between the brains of people who are better at learning their first languages and the brains of people who are worse at learning their first languages	
Determined at birth	A person's ability to learn their first language is determined at birth	
Inheritable	A person inherits their ability to learn their first language from their parents	
Natural ability	People naturally have different levels of ability to learn their first language	
*Multiple Language essentialism*		
Gene	In the future, scientists will be able to determine whether a person is good at learning multiple languages by examining their genes	
Brain	There are consistent differences between the brains of people who easily learn multiple languages and people who struggle to learn multiple languages	
Determined at birth	A person inherits the ability to learn multiple languages from their parents	
Inheritable	A person’s ability to learn multiple languages is determined at birth	
Natural ability	People naturally have different levels of ability in learning multiple languages	

#### Neuromyth belief about bilingualism

The neuromyth questionnaire had 9 items that assessed prevalent neuromyths about bilingualism and bilingual education (the myths were selected based on prior research: Crawford, 1997; Garrity et al., 2019; OECD, 2007; see Table 2 for items). Participants were asked to rate how much they agreed or disagreed with each statement on a 6-point scale for the Prolific sample (1-strongly disagree to 6-strongly agree) and on a 5-point scale for the student sample (1-strongly disagree, 3-neutral, 5-strongly agree). The questionnaire demonstrated high reliability (Cronbach’s alpha = 0.80 for the student and 0.89 for the adult sample).

**Table 2:** Neuromyth belief about bilingualism items

		Item statement
1	Cannot access knowledge across languages	In a bilingual’s mind, knowledge that is acquired in one language is sometimes not accessible in the other language
2	Compete for mental resources	In a bilingual's brain, two languages compete for mental resources
3	Should learn in a sequence	One’s first language should be well-learned before a second language is introduced
4	Cause language problems	Teaching a young child multiple languages can cause language problems such as stuttering or dyslexia
5	Delay language development	Learning two languages simultaneously puts babies at risk for having delayed and possibly impaired language development
6	Confuse children	Exposing children to two languages simultaneously confuses them
7	Impair cognitive ability	Exposing children to two languages impairs their cognitive ability
8	Retained too long in bilingual classrooms	Children in the US who are dual language learners are retained too long in bilingual classrooms
9	Learn at the expense of English acquisition	Children in the US who are dual language learners learn their other language at the expense of English acquisition

## Results and discussion

### Do participants endorse essentialist views regarding language acquisition?

To map out the extent to which individuals hold essentialist views regarding different subtypes of language acquisition, we calculated the means, standard deviations, and proportion agreement for each switched-at-birth and language essentialism questionnaire item. Note that due to experimenter error, all items used a 6-point scale except for the language essentialism questionnaire of the student sample (5-point). Proportion agreement was the number of “*agree*” responses (i.e., somewhat agree, agree, and strongly agree) divided by the total number of responses. For the language essentialism questionnaire from the student sample, we also calculated the proportion of “*neutral*” responses across the students. For all items, ratings did not differ as a function of whether participants self-reported being monolingual vs. being bilingual or multilingual (all *p*s > 0.229, see Supplement 2 for specific t-test results). We next performed a series of *t*-tests between each item and its midpoint (3 for the student questionnaire responses, and 3.5 for the other items, all tests were two-tailed). Itemized results were shown in Tables 3 and 4.

**Table 3:** Study 1 Itemized analysis of the language essentialism questions of the college student sample (N = 386)

	Mean	SD		% Neutral	% Agree	*t* value	*df*	*p* value	Cohen’s* d*
*Switched-at-birth* Answer scale: 1–6, midpoint: 3.5									
Accent	2.21	1.18		NA	17.1	−21.48	385	< .001	1.09
Vocabulary	2.11	1.12		NA	14.0	−24.41	385	< .001	1.24
Grammar	2.01	1.05		NA	10.4	−27.84	385	< .001	1.42
*Average Score*	2.11	1.07		NA	NA	−25.61	385	< .001	1.30
*Language Essentialism Questionnaire* Answer scale: 1–5 (midpoint: 3)									
*Specific Language essentialism*									
Genetically predisposed	2.23	1.02		14.5	15.5	−14.88	385	< .001	0.76
Brain	2.41	1.09		17.4	20.5	−10.62	385	< .001	0.54
Determined at birth	2.32	1.05		17.1	17.8	−12.73	385	< .001	0.65
Inheritable	2.08	0.99		12.7	11.1	−18.30	385	< .001	0.93
*Average score*	2.26	0.89		NA	NA	−16.36	385	< .001	0.83
*First Language essentialism*									
Gene	2.62	1.02		32.6	21.0	−7.35	385	< .001	0.37
Brain	**3.12**	0.89		40.0	37.3	2.59	385	.010	0.13
Determined at birth	2.29	0.96		20.7	14.3	−14.47	385	< .001	0.74
Inheritable	2.65	1.14		22.0	27.2	−6.03	385	< .001	0.31
Natural ability	**3.58**	0.88		22.5	64.0	12.79	385	< .001	0.65
Average Score	2.85	0.62		NA	NA	−4.77	385	< .001	0.24
*Multiple Language essentialism*									
Gene	2.91	0.99		36.5	30.8	−1.69	385	.092	0.09
Brain	**3.48**	0.84		30.6	57.5	11.36	385	< .001	0.58
Determined at birth	2.22	0.88		20.5	9.8	−17.56	385	< .001	0.89
Inheritable	2.51	0.97		27.7	17.9	−9.99	385	< .001	0.51
Natural ability	**3.77**	0.93		17.6	72.3	16.31	385	< .001	0.83
*Average score*	2.98	0.62		NA	NA	−0.67	385	0.503	0.03

See Table 1 for specific descriptions of items. The “genetically predisposed” and “gene” items tap into the same aspect of essentialism (i.e., “innateness”) but with slightly different wordings. T-tests are one-sample independent tests against midpoints. Items significantly greater than the midpoint are bolded. Items significantly greater than midpoints are bolded

**Table 4:** Study 1 Itemized analysis of the language essentialism Questions of the prolific adult sample (N = 340)

	Mean	SD	% Agree	*t* value	*df*	*p* value	Cohen’s* d*
*Switched-at-birth* Answer scale: 1–6, midpoint: 3.5							
Accent	2.24	1.21	16.8	−19.28	339	< .001	1.05
Vocabulary	2.19	1.17	14.4	−20.51	339	< .001	1.11
Grammar	2.18	1.12	15.0	−21.79	339	< .001	1.18
*Average Score*	2.20	1.11	NA	−21.58	339	< .001	1.17
*Language Essentialism Questionnaire* Answer scale: 1–6 (midpoint: 3.5)							
*Specific Language essentialism*							
Genetically predisposed	2.35	1.37	19.7	−15.45	339	< .001	0.84
Brain	2.56	1.47	26.2	−11.85	339	< .001	0.64
Determined at birth	2.44	1.35	24.4	−14.42	339	< .001	0.78
Inheritable	2.41	1.41	22.4	−14.19	339	< .001	0.77
*Average Score*	2.44	1.25	NA	−15.64	339	< .001	0.85
*First Language essentialism*							
Gene	2.83	1.30	32.9	−9.52	339	< .001	0.52
Brain	3.57	1.16	58.2	1.12	339	0.264	0.06
Determined at birth	2.52	1.27	22.9	−14.17	339	< .001	0.77
Inheritable	3.07	1.55	41.8	−5.08	339	< .001	0.28
Natural ability	**4.28**	1.23	78.5	11.73	339	< .001	0.64
Average Score	3.25	.87	NA	−5.19	339	< .001	0.28
*Multiple Language essentialism*							
Gene	3.24	1.22	47.9	−3.96	339	< .001	0.21
Brain	**3.94**	1.02	73.2	8.00	339	< .001	0.43
Determined at birth	2.55	1.18	20.3	−14.84	339	< .001	0.80
Inheritable	2.91	1.26	37.4	−8.55	339	< .001	0.46
Natural ability	**4.41**	1.14	85.3	14.83	339	< .001	0.80
*Average score*	3.41	.79	NA	−2.06	339	0.039	0.11

See Table 1 for specific descriptions of items. The “genetically predisposed” and “gene” items tap into the same aspect of essentialism (i.e., “innateness”) but with slightly different wordings. T-tests are one-sample independent tests against the midpoint. Items significantly greater than the midpoint are bolded

For the switched-at-birth and the specific language essentialism questions, participants in general held relatively low levels of essentialist views, with some variation across items (Tables 3 and 4). All items were significantly below the midpoint. For the switched-at-birth items, people generally *disagreed* that learning the language of one’s birth parents was biologically determined, and this held for different aspects of the language, including accent, vocabulary, and grammar. For the specific language essentialism items, people overall *disagreed* that learning the language of one’s birth parents was genetically predisposed, determined at birth, or inherited from their birth parents, or that their brain was naturally tuned to learn the language. However, there were still sizable proportions of participants who had specific language essentialism views (10.4–26.2% were above midpoints across items and samples).

Unlike the specific language essentialism subscale in which all items were consistently below the midpoints, responses to the first and multiple language essentialism questions varied greatly across items. Specifically, participants overall endorsed that there are brain differences between those who learn multiple languages more easily vs. with more difficulty. The students but not adults reported that such brain differences also exist between those who learn their first language more easily vs. with more difficulty. For the *natural ability* item, participants on average agreed that people naturally differ in their ability to acquire language as well as to acquire multiple languages. In contrast, participants overall disagreed that the ability to acquire language or multiple languages is detectable in the genes, determined at birth, or inheritable.

### How is language essentialism associated with believing in neuromyths about bilingualism?

To characterize the extent to which participants believe in each neuromyth statement, we mapped out the means, standard deviations, and proportion agreement for each item (Table 5). To test how language essentialism might be associated with endorsement of neuromyths about bilingualism, we conducted a series of correlational analyses between the neuromyth beliefs and the specific language essentialism, first language essentialism, and multiple language essentialism subscales, respectively. Average scores were used to represent a comprehensive essentialist reasoning for each aspect of language acquisition. Gender and age were entered as control variables. For the Prolific adult sample, education level was also entered as a control. Table 6 displays the partial correlation results.

**Table 5:** Study 1 Itemized analysis of the neuromyth statements

	Item	College student sample (*N* = 386)				Prolific adult sample (*N* = 340)		
		Mean	SD	% Neutral	% Agree	*Mean*	*SD*	% Agree
1	Cannot access knowledge across languages	3.27	1.04	27.7	49.2	3.14	1.29	42.7
2	Compete for mental resources	2.38	.93	24.9	13.7	2.56	1.25	24.4
3	Should learn in a sequence	2.44	1.06	21.2	17.1	2.82	1.44	30.9
4	Cause language problems	1.92	.83	16.1	4.2	2.00	1.01	8.8
5	Delay language development	1.94	.86	16.1	5.4	2.09	1.16	12.9
6	Confuse children	2.27	.97	11.7	13.0	2.37	1.24	20.3
7	Impair cognitive ability	1.71	.81	7.8	3.9	1.97	1.13	10.6
8	Retained too long in bilingual classrooms	2.61	.81	48.7	10.1	2.60	1.10	20.0
9	Learn at the expense of English acquisition	2.15	.95	18.9	9.8	2.31	1.26	18.8

Answer scale: 1–5 for the student and 1–6 for the adult samples

**Table 6:** Study 1 Partial correlation between language essentialism and neuromyth endorsement

	1	2	3	4	5
1. Neuromyth belief endorsement		.24***	.27***	.18***	.10*
2. Switched-at-birth	.37***		.63***	.35***	.27***
3. Specific Language essentialism	.43***	.56***		.47***	.34***
4. First Language essentialism	.42***	.37***	.54***		.53***
5. Multiple Language essentialism	.36***	.33***	.42***	.65***	

Student data are above the diagonal (*N* = 386), controlling for age, gender, and bilingual status; Prolific adult data are below the diagonal (*N* = 340), controlling for age, gender, bilingual status, and education. All correlations were significant at ***p < .001 except for the *p < .05

Across the two samples, switched-at-birth and specific language essentialism were both positively moderately correlated with endorsing neuromyth beliefs about bilingualism (all *r*s = 0.24–0.43, all *p*s < 0.001), when controlling for age, gender, bilingual status, and education (education only applied to the Prolific sample). Therefore, participants who held a biological account for learning the language of one’s birth parents were also more likely to hold false beliefs about bilingualism. In addition, neuromyth beliefs were also moderately associated with first and multiple language essentialism views in the Prolific adult sample (*r*s = 0.42 and 0.36, *p*s < 0.001), but the associations were weak in the student sample (*r* = 0.18, *p* < 0.001, and *r* = 0.10, *p* < 0.05, respectively).

## Study 2

Study 1 revealed individual differences in people’s essentialist reasoning about different subtypes of language acquisition. Importantly, across two samples, endorsing a biological account for learning the language of one’s birth parents was associated with misconceptions about multilingual acquisition. Study 2 aimed to examine another important potential consequence of holding essentialist reasoning about language acquisition: policy endorsement of bilingual education. Study 2 hypothesized that essentialist views on language acquisition are significantly associated with policy endorsement beliefs about bilingual education. Specific study design, hypotheses, and analyses were pre-registered (As. Predicted anonymous link https://aspredicted.org/P8Q_R8R).

## Method

### Participants

Participants included *N* = 289 adults recruited from Prolific. To be eligible for participation, participants were required to (1) use a US IP address, and (2) be monolingual English-speaking adults. The second criterion was applied to ensure a sample with homogenous language experiences. Participants completed a survey and were paid $3.00. Their demographic information was as follows: age, *M* (*SD*) = 31.37 (12.14); gender, 55.7% female, 42.6% male, 1.7% non-binary; race, 87.2% white, 4.8% Black, 4.5% Latinx, 2.1% Asian, 0.7% Native American, and 0.7% Native Hawaiian or Pacific Islander; 49.1% holding at least a bachelor’s degree. Of the final sample, *N* = 46 were recruited in a second round to collect politically conservative participants, as participants from the first round were disproportionately liberal (72.4% of the first-round participants). The second round of data collection was identical to the first round, except that only participants who self-identified as politically conservative were eligible to enter the survey (i.e., “Where would you place yourself along the political spectrum?”, “conservative”). The final sample had 38.8% and 61.2% participants who identified themselves as conservatives and liberals, respectively. Moreover, 81% of participants reported having experiences learning a non-English language. An additional *N* = 25 participants were removed because they failed to pass all attention check items. Data will be available on OSF upon publication.

### Procedure

Participants completed a questionnaire with five blocks. The first three blocks were identical to those in Study 1. Block 4 measured participants’ bilingual policy endorsement. Block 5 was similar to Study 1 block four but with several additional questions asking for participants’ political orientation and language learning experiences. The survey had a fixed order of all blocks, and the order of items was randomized within each block or subscale. The bilingual policy endorsement survey can be found in Supplement 1.

### Measures

#### Bilingual policy endorsement

Bilingual policy endorsement was assessed by a 10-item questionnaire measuring attitudes regarding different aspects of bilingualism, such as financial investments in bilingual education, bilingual school instruction, and bilingual curriculum. The items were developed based on prior published research and official government-conducted surveys (Canadian Commissioner of Official Languages, 2021; Krashen, 1996; Shin & Krashen, 1996). Participants rated how much they agreed or disagreed with each statement on a 6-point scale (1-strongly disagree to 6-strongly agree). Items showed very high reliability (Cronbach’s alpha = 0.91). A higher score indicates an endorsement of policies that *oppose* bilingualism.

#### Political orientation

Political orientation was indicated by a single item, “Please indicate your political orientation”, on a 6-point scale (1-strongly liberal to 6-strongly conservative). A higher score indicates a more conservative orientation.

#### Non-English language learning experience

Participants’ non-English language learning experience was measured by a single item (“Have you had experiences learning a non-English language?”) with a binary response (yes or no).

## Results and discussion

### Language essentialism endorsement

Similar to Study 1, the switched-at-birth items and the language essentialism questionnaire subscales all demonstrated high internal consistencies, Cronbach’s alphas = 0.94 and 0.87, respectively. Table 7 displays the means, standard deviations, and proportion agreement for each item, as well as the independent sample t-tests against the midpoint. These results replicated what was found in Study 1. First, participants generally disagreed with the essentialist idea that learning the language of one’s birth parents is biologically based, yet there was some variation (11.1–29.4% holding essentialist views). Second, participants endorsed the essentialist ideas that first language acquisition and multiple language acquisition rely on natural abilities, and that individuals’ brains are consistently different if they easily acquire multiple languages (60.6–85.1% holding essentialist views). In contrast, participants overall disagreed with essentialist attitudes that acquiring the first language and acquiring multiple languages are detectable in the genes, determined at birth, or inheritable (20.4–48.8% holding essentialist views).

**Table 7:** Study 2 Itemized analysis of the language essentialism questions (N = 289)

	Mean	SD	% Agree	*t* value	*df*	*p* value	Cohen’s* d*
*Switched-at-birth*							
Accent	2.10	1.21	15.2	−19.77	288	< .001	1.16
Vocabulary	1.97	1.15	11.8	−22.70	288	< .001	1.34
Grammar	1.94	1.08	11.1	−24.48	288	< .001	1.44
*Average Score*	2.00	1.09	NA	−23.43	288	< .001	1.38
*Language Essentialism Questionnaire*							
*Specific Language essentialism*							
Genetically predisposed	2.37	1.32	19.7	−14.54	288	< .001	0.86
Brain	2.71	1.47	29.4	−9.10	288	< .001	0.54
Determined at birth	2.62	1.43	26.6	−10.48	288	< .001	0.62
Inheritable	2.33	1.34	20.4	−14.80	288	< .001	0.87
*Average Score*	2.79	.96	NA	−12.71	288	< .001	0.75
*First Language essentialism*							
Gene	3.09	1.31	42.6	−5.27	288	< .001	0.31
Brain	3.63	1.23	60.6	1.79	288	0.075	0.11
Determined at birth	2.65	1.27	27.3	−11.42	288	< .001	0.67
Inheritable	3.13	1.50	44.6	−4.15	288	< .001	0.24
Natural ability	**4.25**	1.22	79.6	10.49	288	< .001	0.62
*Average Score*	3.35	.92	NA	−2.76	288	0.006	0.16
*Multiple Language essentialism*							
Gene	3.27	1.22	48.8	−3.17	288	< .001	0.19
Brain	**4.07**	1.12	76.1	8.71	288	< .001	0.51
Determined at birth	2.37	1.17	20.4	−16.34	288	< .001	0.96
Inheritable	2.82	1.26	36.0	−9.12	288	< .001	0.54
Natural ability	**4.44**	1.20	85.1	13.29	288	< .001	0.78
*Average score*	3.40	.85	NA	−2.09	288	0.038	0.12

All answers followed a 1–6 scale; T-tests are one-sample independent tests against the midpoint. Items significantly greater than the midpoint are bolded

### Pre-registered Hypothesis 1: Language essentialism is associated with believing neuromyths about bilingualism.

Our pre-registered hypothesis 1 aimed to test whether language essentialism reasoning predicts neuromyth endorsement, as in Study 1. Table 8 displays the means, standard deviations, and proportion agreement for each neuromyth item, and the scale again demonstrated high overall reliability (Cronbach’s alpha = 0.86). Table 9 displays the bivariate correlations among the scales. Along with Study 1, the correlation results showed that overall language essentialism beliefs were moderately associated with neuromyth beliefs, *r*s = 0.26–0.49, *p*s < 0.001. Table 10 displays the partial correlations between neuromyth beliefs and language essentialism, controlling for age, gender, education, and non-English learning experience. These associations remained significant after regressing out the control variables, *r*s = 0.26–0.49, *p*s < 0.001. Thus, consistent with Study 1 findings, language essentialism subscales were reliably associated with neuromyth endorsement. In other words, those who were more likely to endorse a biological account for language acquisition were also more likely to believe in neuromyths about bilingual acquisition.

**Table 8:** Study 2 Itemized analysis of the neuromyth statements

	Item	Study 2 Sample (*N* = 289)		
		Mean	SD	% Agree
1	Cannot access knowledge across languages	3.22	1.21	48.1
2	Compete for mental resources	2.56	1.16	22.5
3	Should learn in a sequence	2.69	1.39	29.1
4	Cause language problems	1.84	1.05	7.3
5	Delay language development	2.07	1.12	12.1
6	Confuse children	2.24	1.19	18.0
7	Impair cognitive ability	1.81	1.09	8.0
8	Retained too long in bilingual classrooms	2.49	1.02	13.8
9	Learn at the expense of English acquisition	2.14	1.10	12.8

Answer scale: 1–6

**Table 9:** Bivariate correlations among language essentialism subscales, neuromyth, and policy endorsement

	1	2	3	4	5	6	7
1. Non-English learning experience							
2. Political orientation	−.19**						
3. Switched-at-birth	−.27***	.23***					
4. Specific language essentialism	−.22***	.31***	.45***				
5. First language essentialism	−.16**	.17**	.33***	.37***			
6. Multiple language essentialism	−.12*	.21***	.22***	.30***	.75***		
7. Neuromyth endorsement	−.27***	.39***	.39***	.49***	.33***	.26***	
8. Policy endorsement	−.33***	.59***	.28***	.26***	.14*	.13*	.44***

*p < .05; **p < .01; ***p < .001

**Table 10:** Partial correlations among language essentialism subscales, neuromyth, and policy endorsement

	1	2	3	4	5
1. Adapted switched-at-birth					
2. Specific language essentialism	.45***				
3. First language essentialism	.33***	.37***			
4. Multiple language essentialism	.22***	.30***	.75***		
5. Neuromyth endorsement	.39***	.49***	.33***	.26***	
6. Policy endorsement	.28***	.25***	.14*	.13*	.44***

*p < .05; ***p < .001. All correlations controlled for age, gender, non-English learning experience, and education. Political orientation was controlled in all correlations with policy endorsement (the last row)

To find out which subtypes of language essentialism better predict neuromyth beliefs, we next conducted a series of comparisons among these correlation coefficients. Analyses were done with the R package “*cocor*”, function “*cocor.dep.groups.overlap*”, which applies methods based on Fisher’s r-to-z transformation to conduct significance tests between correlation coefficients with overlapping variables and overlapping samples. Results showed that, after partialling out the control variables, specific language essentialism had a stronger correlation with neuromyth beliefs, compared to first language essentialism, *z* = 2.76, *p* = 0.006, and multiple language essentialism, *z* = 3.71, *p* < 0.001, but not switched-at-birth items, *z* = 1.87, *p* = 0.062. There were no significant differences in the associations with neuromyth beliefs, among switched-at-birth items, first language essentialism, and multiple language essentialism measures, *z*s = 0.97–1.92, *p*s = 0.054–0.332. Therefore, specific language essentialism, or endorsing a biological account for learning the language of one’s birth parents, was *more* reliably associated with neuromyth endorsement than other aspects of language essentialism.

### Pre-registered Hypothesis 2: Language essentialism is associated with opposing policies promoting bilingual education

The pre-registered hypothesis 2 aimed to test whether language essentialism reasoning predicts bilingual policy endorsement. Note that after reverse-coding for reverse items, a higher score indicated opposing the policy. As shown in Table 9, overall, language essentialism beliefs had small-to-medium, but significant associations with policy endorsements, *r*s = 0.13–0.28, *p*s < 0.05. Table 10 displays the partial correlations between policy endorsements and language essentialism, controlling for age, gender, education, non-English learning experience, and political orientation. Associations remained similarly after regressing out the control variables, *r*s = 0.13–0.28, *p*s < 0.05. In sum, our data showed a small but significant effect that participants who endorsed a more biological account for language acquisition were more likely to oppose educational policies promoting bilingual education.

To find out which subtypes of language essentialism better predict policy beliefs, we again conducted a series of comparisons among these correlation coefficients with the R package “*cocor*” and function “*cocor.dep.groups.overlap*” (same as in Study 1). Results showed that the switched-at-birth items had stronger associations with policy endorsements compared to first language essentialism, *z* = 2.13, *p* = 0.033, and multiple language essentialism, *z* = 2.12, *p* = 0.034, but not specific language essentialism, *z* = 0.51, *p* = 0.610. Specific language essentialism was not more strongly associated with policy endorsements as compared to first language essentialism, *z* = 1.72, *p* = 0.086; multiple language essentialism, *z* = 1.78, *p* = 0.076. No differences were found between first language and multiple language essentialism in their associations with policy endorsement, *z* = 0.24, *p* = 0.808. Therefore, results revealed that those believing that one inherits the ability to learn the language of their birth parents were *more* likely to oppose educational policies promoting bilingual education.

## General discussion

Although there have been debates about the nature of language acquisition among both scientists and lay individuals (Kidd et al., 2018; Moin et al., 2013; Patterson, 2020; Wulff & Ellis, 2018), little research has been conducted to directly characterize how lay people reason about language acquisition. The present research used psychological essentialism as a lens to characterize people’s reasoning. We first developed survey items to capture different essentialist beliefs (e.g., innateness, heritability) for three subtypes of language acquisition (i.e., specific, first, and multiple language acquisition). Next, we tested how language essentialism might be related to two belief outcomes: language-related neuromyths and educational policy endorsements. Specific findings and implications are discussed below.

### Characterizing how people reason about language acquisition subtypes with psychological essentialism

The present two studies indicate that how people think about different subtypes of language acquisition indeed differs. As a reminder, the two essentialism measures were our switched-at-birth questions and language essentialism scales. Both of these measures tapped into the belief that one is predisposed to speak either (1) a specific language, (2) one’s first language or (3) multiple languages because of something innate, biologically based, and inherited from birth parents. Across Studies 1 and 2, we found that participants were most essentialist about the subtype of multiple language acquisition, followed by first language acquisition and specific language acquisition.

Diving deeper into our findings, results across studies showed relatively low levels of essentialism endorsement for specific language acquisition, with all items significantly below midpoints. In contrast, participants were considerably more likely to essentialize first and multiple language learning (see also Lou & Noels, 2016 for comparable patterns in mindset beliefs about language learning). The low levels of essentialism regarding specific language acquisition are not surprising, given that people acquire the language of their childhood family and community (e.g., a child exposed to Swahili will learn Swahili, regardless of their ethnicity). However, some participants did endorse language essentialist reasoning. For example, more than one-fourth of participants reported that a predisposition to learn a specific language is *determined at birth* and reflected in one’s *brain*. These findings are consistent with prior anecdotal observations that some people may tie an individual’s ethnicity with their propensity to learn a certain language. For example, Kinzler (2020) reported anecdotes of some people suggesting that “Jewish students innately outperform others in a Hebrew class.” Our results offered empirical support that, despite scientific evidence that infants are equally able to learn any language as long as they are exposed to it (Werker & Hensch, 2015), a sizeable subset of people still believe that one tends to better acquire the language of the socio-cultural background of one’s biological parents (i.e., family, community, ethnicity).

We note that the switched-at-birth task referred to a language (Korean) that is correlated with a nationality that differed from that of the adoptive parents, and with ethnicity and race that may have been assumed to differ from that of the adoptive parents. Languages vary in terms of how they relate to nationality, ethnicity, and race, and thus an important open question is whether people’s judgments may be sensitive to such factors. For example, when languages are believed to correspond to other important social identities (e.g., nationality, ethnicity, race), participants may hold stronger essentialist interpretations about how such languages are learned. This remains an important question for future research.

Moreover, the switched-at-birth task taps into a similar construct as the specific language essentialism subscale. Indeed, results demonstrated moderate correlations between the two (Tables 6 and 9). At the same time, these two measures tap into different components of essentialism, with the switched-at-birth (SWAB) task focused more directly on inheritance. The specific language essentialism subscale assesses a broader range of essentialist reasoning (e.g., genetic basis, neural basis).

### Language essentialism, neuromyth, and policy endorsements

Our results revealed that holding an essentialist interpretation of language had important implications for real-world educational issues. In particular, individuals expressing higher levels of language essentialism were more likely to endorse neuromyths about bilingual acquisition (Studies 1 and 2). These individuals were also less likely to endorse educational policies promoting multilingualism (Study 2). Moreover, the subtypes of language essentialism were differentially associated with these belief outcomes. Interestingly, specific language essentialism, or endorsing a biological account in learning the language of one’s biological parents, was a stronger predictor of both outcomes, compared to first and multiple language essentialism. Why this is the case is currently unknown, but these beliefs may reflect a lack of knowledge about language and learning more generally, which then is expressed both in essentialist reasoning (especially towards the specific language of one’s birth parents) and neuromyth endorsement. In prior work, we have found a parallel association between essentialist beliefs and neuromyths in the domain of learning styles (Nancekivell et al., 2020, 2021). Future research should examine how psychological essentialism may moderate other neuromyth endorsements. For example, perhaps essentializing intellectual abilities corresponds to endorsing neuromyths about learning impairments or differences.

The present work also has implications for people’s endorsement of policies related to bi-/multi-lingual education. As noted, we found that higher levels of language essentialism were associated with opposing educational policies for bilingualism (Study 2). For example, those who are more likely to endorse an essentialist account of language acquisition were more likely to embrace English-only instruction and oppose bilingual education at school. In other words, people’s different stances on bilingualism and their choices on bilingual education may be in part explained by whether they hold essentialist beliefs about how children acquire languages. In a broader sense, these results may also reflect long-standing educational and policy debates on bi-/multi-lingual education, such as whether bi-/multi-lingual education is detrimental to children’s cognitive development and/or educational careers (Hakuta, 1999; Ovando, 2003). Future work could explore the relation between holding essentialist reasoning about language acquisition and teaching practices surrounding language. For example, prior work suggests some teachers try to maintain monolingual norms in their classes, while others flexibly use different languages to facilitate learning (Cekaite & Evaldsson, 2008; Chimirala, 2017; de Oliveira et al., 2016). We propose that the survey employed in the present study might be able to capture important variability in teachers’ beliefs and thereby predictors of these practices.

Future studies could also use the current measures to probe potential cross-cultural differences in these beliefs. The current samples were limited to the US context, where most individuals are English monolinguals. Therefore, in countries where multilingualism is more common, individuals may hold different views about language acquisition. For example, these populations might essentialize multilingualism less as it is more common.

## Conclusion

The current study is the first that characterizes lay theories about language acquisition and its association with broader beliefs about education. Using a psychological essentialism framework, Study 1 revealed individual differences in how lay individuals reason about different aspects of language acquisition—namely, individuals were the most essentialist toward learning a specific language, followed by learning one’s first language and learning multiple languages. Importantly, Studies 1 and 2 showed that language essentialism is associated with endorsing educational neuromyths and opposing policies promoting bilingual education. Together, the current research contributes to the literature in understanding the complexity of people’s intuitive theories about learning, and results have important implications for cognitive research in the context of education.

## Data Availability

Data and code are available on OSF: https://osf.io/m34gh/?view_only=fe3484a422244ef19d75d53e0da05f3c.
